# Management of severe childhood pneumonia by day care approach in developing countries

**DOI:** 10.15171/hpp.2018.11

**Published:** 2018-01-07

**Authors:** Yasmin Jahan, SM Atiqur Rahman, Abu Sayeed Chowdhury, Md Moshiur Rahman

**Affiliations:** ^1^Graduate School of Biomedical & Health Sciences, Hiroshima University, Japan; ^2^School of Health, University of New England, Australia

**Keywords:** Day Care Approach, Developing countries, Severe childhood pneumonia

## Abstract

**Background:** Pneumonia is a major cause of child mortality among children under 5 years, worldwide. Pneumonia infection may be caused by bacteria, viruses, or fungi in single or in both lungs. According to recent criteria developed by the World Health Organization(WHO) in September (2013), pneumonia can be classified into severe pneumonia, pneumonia and no pneumonia. Most of the deaths occur from severe pneumonia.

**Methods: **Disease management of severe childhood pneumonia requires early identification,prompt referral and the availability of intensive quality care. Under 5 years old children with severe pneumonia should receive day care, with antibiotic treatment, feeding, and supportive care with similar 24-hour hospital treatment.

**Results:** Considering that difficulties, International Centre for Diarrheal Disease Research,Bangladesh (ICDDR, B) initiated Day Care Approach (DCA) model, as an innovative, safe,effective and less expensive alternative to hospital management of severe childhood pneumonia.A 24 months old girl came to the health care center with severe breathing difficulty, cough,history of fever and head nodding. The management described below was continued daily until there was clinical improvement; no fever, no fast breathing, no lower chest wall in drawing, no danger signs, no rales on auscultation, and no hypoxemia.

** Conclusion:** Considering the WHO case management protocol for severe pneumonia, DCA recommends that diagnosis of severe pneumonia should be based primarily on visible clinical parameters. On that basis, severe childhood pneumonia can be successfully managed at daycare clinics including for children with hypoxemia who is required prolong (4-6 hours) oxygen therapy.

## Introduction

Globally, Pneumonia sustains as the leading cause of death among under 5 years old children.^1^ The recent World Health Organization (WHO) global report (2013) deemed that pneumonia accounts for approximately 120 million cases every year,^2^ among which 14 million (12%) progress to severe pneumonia^3^; and developing countries belong into the most vulnerable vicinity (95%).^1^ Reported deaths in a year of this age group was 0.9 million which represents about 17% of all deaths among children under 5.^4^ More than 99% of all pneumonia deaths occur in low- and middle-income countries (LMIC). For instance, South Asia and sub-Saharan Africa, specifically, suffer more than two-thirds of worldwide pneumonia burden; 13% of which covers children from Bangladesh.^5^

According to the WHO guideline, successful management of severe childhood pneumonia requires hospitalization for supportive treatment, such as suctioning, oxygen therapy, fluid and nutritional management, and close monitoring.^6-9^ In Bangladesh, inadequacy of pediatric hospital beds for severe pneumonia patients is a major challenge. A prospective observational study has resulted that, day care facility based modified primary care management for severe pneumonia is more successful and cost-effective as an alternative in respect to hospitalization.^10^ Previous research indicated positive outcomes (both efficacy and safety) of a day care-based management. The International Centre for Diarrheal Disease Research, Bangladesh (ICDDR, B), by following the outcomes, developed an innovative model of Day Care Approach (DCA) as a safer and less expensive alternative to hospital management of severe childhood pneumonia. The fundamental theme of this model urges initiating patient management in the community for those severe patients who can not be hospitalized. This will be applicable for both in the urban outpatient clinics, such as Comprehensive Reproductive Health Centres (CRHCs) and in the rural clinics such as Health & Family Welfare Centres (HFWCs). As a consequence, DCA has become a validated approach to free up hospital beds in LMIC.^11^

## Case Report

A 2 years old female child came from the rural site of Bangladesh and presented with a history of breathing difficulty, cough and fever for two days. Her physical examination revealed the past case history of grunting, very severe chest wall indrawing and hypoxemia (SPO2 84% without O2) and head nodding. She was clinically diagnosed with severe pneumonia case. On admission, her temperature was 37.4^○^C, RR 73 breaths/min, PR 174 beats/min, and SPO2 was 84% without O2. Patient was Dyspneic and irritated. On Auscultation, crepitation was present in both lung fields and rhonchi was present on upper and middle side of left lung field. She was admitted in a Health and Family Welfare Centre under the DCA basis and treated as a severe pneumonia patient immediately, an out-patient facility providing space, trained staff members (doctors, nurses), beds, and the necessary equipment’s like antibiotics, oxygen therapy, nebulization, nasopharyngeal suction etc. as indicated.

Treatment and diet that the patient received (according to the body weight):

1. Diet- Breast feeding + Milk Suji 12 mL/kg/feed
2. Oxygen inhalation: 2 L/min - Stat and SOS
3. Inj. Ceftriaxone: 1 gm I/M once daily for 5 days
4. Syp. Levosalbutamol: 1 ½ TSF P/O 12 hourly for 7 days
5. Syp. Paracetamol: 1 TSF P/O 8 hourly (for fever more than or equal to 38^◦^C)

No investigations were done due to inadequate facilities.

### Expected outcome of the treatment plan

Clinical improvement of the patient; no fever, no hypoxemia, no fast breathing and no tachycardia after the fifth day of treatment.

### Actual outcome

On day 0 (admission day): The patient was diagnosed with following physical conditions: age specific fast breathing, severe lower chest wall indrawing, grunting and hypoxemia, temperature (37.4^◦^C, not fever), respiratory rate (73 breaths/min), SPO2 (84%; without O2) and pulse rate (174 beats/min). SPO2 improved after providing 5 hours of continuous Oxygen supply. With provision of O2, SPO2 was 95%, 89%, 93% and 94% respectively at the beginning and after 2 minutes, 15 minutes and 1 hour of removing O2. Respiratory rate was 50 breaths/min, temp was 37^◦^C.

On day 1: There was age specific fast breathing, tachycardia and chest wall indrawing. Temperature was 36.9^◦^C, Respiratory rate was 42 breaths/min, SPO2 was 96% without O2 though the child was kept without O2 for overnight and pulse rate was 155 beats/min.

On day 2: There was mild chest indrawing but no age specific fast breathing, no fever and no hypoxemia. Temperature was 36.1^◦^C, respiratory rate was 38 breaths/min, SPO2 was 96% without O2 and pulse rate was 134 beats/min.

On day 3: Patient started improving clinically but there was age specific fast breathing but no chest indrawing, no fever, no tachycardia and no hypoxemia. Temperature was 36^◦^C. Respiratory rate was 41 breaths/min, SPO2 was 100 % without O2 and pulse rate was 130 beats/min.

On day 4: Patient was clinically improved and there was no hypoxemia, no fever, no age specific fast breathing and no tachycardia. Temperature was 36.8^◦^C. Respiratory rate was 36 breaths/min, pulse rate was 134 beats/min, SPO2 was 99% without O2.

On day 5: Patient was clinically stable, and her temperature was 36.6^◦^C. Respiratory rate was 34 breaths/min, SPO2 was 100% without O2 and pulse rate was 116 beats/min (Table 1).

As the child was clinically improved and there was no fever, no hypoxemia, no fast breathing and no tachycardia, she was discharged from Health and Family Welfare Centre.

## Discussion

Increasing awareness of the health problems is probably having significant advantages for clinical and public health domains. The seamless and reliable detection of hypoxemia could be accomplished through the extended utilization of pulse oximetry, which is a non-invasive measure of arterial oxygen saturation. To do so, oxygen treatment must be more generally accessible in numerous remote settings. The proper utilization of oxygen concentrators will be an effective way that can run on regular or as an alternative source of power.

Hypoxemia is hardly well recognized or managed in mal-allocated settings even though it has a great importance in all types of acute severe illness. Still oxygen treatment remains an inaccessible luxury for a large proportion of severely ill children admitted to hospitals in developing countries. This is particularly true for patients in small district hospitals, where, even if some facility for delivering oxygen is available, supplies are often unreliable and the benefits of treatment may be diminished by poor maintenance, faulty equipment’s, poorly trained staff and inadequate guidelines.^12^ Many developing countries have gradually been experiencing in the clinical, organizational, biomedical technology and training aspects of setting up and sustaining effective oxygen delivery systems in hospitals and small healthcare facilities. A surprising but strong evidence indicates that, use of pulse oximetry and the availability of reliable oxygen sources in district and provincial hospitals can reduce pneumonia related death rates up to one third.^13^

The main purpose of DCA is to assess the effectiveness of severe childhood pneumonia treatment on a day care basis as well as to quantify the cost-effectiveness compare to hospitalization. This case management proved that, children with severe pneumonia can be treated on a day care basis, as effectively as in the hospital. Moreover, the study findings may have a great impact on the treatment of severe childhood pneumonia, particularly in countries with poor resources and limited hospital beds. Therefore, the case management can be suggested for replication in most urban and rural outpatient clinics in developing countries through providing necessary training, logistic support and motivation to the staff members.

## Conclusion

DCA may be a feasible mode of applying scarce hospital beds in developing countries more efficiently by selecting day care treatment for children with severe pneumonia. The solution could be implemented followed by the WHO guideline in those hospitals which have been identified as requiring hospitalization. This practical approach would be effective for both developing and other developed and/or underdeveloped countries where similar health resources and infrastructures are available. In this regard, the health care providers would be familiar with clinical symptoms in order to understand the presence of danger signs of pneumonia like hypoxemia. This case study suggests for upgrading the existing day care facilities by promoting training to service providers, procurement of modern equipment and create friendly and secure atmosphere for the customers. Moreover, integrating DCA in health promotion strategy will benefit to develop new healthcare policy and particularly the cost-effectiveness will assist to increase consumer interest.

## Ethical Approval

Written informed consent was taken from the patient.

## Competing interests

The authors declare that they have no competing interests.

## Funding

No funding was used to conduct this report or to prepare this case report.

## Authors’ contributions

YJ wrote the initial text. MM, SAR, ASC and MMR reviewed and complemented it. All authors critically reviewed the manuscript and approved the final version submitted for publication.

## Acknowledgements

I would like to express my appreciation to my co-authors for their full-time cooperation.

**Table 1:** Clinical evaluation during day care approach

**Day**	**Clinical features**				
	**Respiratory rate/min**	**Temperature**	**Pulse rate/min**	**O**_2_	**Lungs findings**
Day-0	73	37.4	174	84% without O_2_ (O_2_ continues 5 hours) with provision of O_2_, SPO_2_ was 95%, 89%, 93% and 94%	Crepitation & Rhonchi present in both lung (B/L) field
Day-1	42	36.9	155	96% without O_2_	Crepitation present and rhonchi resolved by nebulization (B/L)
Day-2	38	36.1	134	96% without O_2_	Crepitation present (B/L)
Day-3	41	36.0	130	100% without O_2_	Crepitation present right lung field
Day-4	36	36.8	134	99% without O_2_	Crepitation present at right lung field
Day-5	34	36.6	116	100% without O_2_	No crepitation and Rhonchi present
